# Analysis of Circulating miRNA Profile in Plasma Samples of Glioblastoma Patients

**DOI:** 10.3390/ijms22105058

**Published:** 2021-05-11

**Authors:** Dóra Géczi, Bálint Nagy, Melinda Szilágyi, András Penyige, Álmos Klekner, Adrienn Jenei, József Virga, Zsuzsanna Birkó

**Affiliations:** 1 Department of Human Genetics, Faculty of Medicine, University of Debrecen, 4032 Debrecen, Hungary; g.dora@med.unideb.hu (D.G.); nagy.balint@med.unideb.hu (B.N.); szilagyi.melinda@med.unideb.hu (M.S.); 2 Department of Human Genetics, Faculty of Medicine, Faculty of Pharmacy, University of Debrecen, 4032 Debrecen, Hungary; penyige@med.unideb.hu; 3 Department of Neurosurgery, Faculty of Medicine, University of Debrecen, 4032 Debrecen, Hungary; klekner.almos@med.unideb.hu (Á.K.); jenei.adrienn@med.unideb.hu (A.J.); 4 Department of Oncology, Faculty of Medicine, University of Debrecen, 4032 Debrecen, Hungary; virga.jozsef@med.unideb.hu

**Keywords:** glioblastoma, circulating miRNA, blood plasma, NanoString, network analysis, biomarker

## Abstract

(1) Background: Glioblastoma multiforme (GBM) is among the most aggressive cancers with a poor prognosis. Treatment options are limited, clinicians lack efficient prognostic and predictive markers. Circulating miRNAs—besides being important regulators of cancer development—may have potential as diagnostic biomarkers of GBM. (2) Methods: In this study, profiling of 798 human miRNAs was performed on blood plasma samples from 6 healthy individuals and 6 patients with GBM, using a NanoString nCounter Analysis System. To validate our results, five miRNAs (hsa-miR-433-3p, hsa-miR-362-3p, hsa-miR-195-5p, hsa-miR-133a-3p, and hsa-miR-29a-3p) were randomly chosen for RT-qPCR detection. (3) Results: In all, 53 miRNAs were significantly differentially expressed in plasma samples of GBM patients when data were filtered for FC 1 and FDR 0.1. Target genes of the top 39 differentially expressed miRNAs were identified, and we carried out functional annotation and pathway enrichment analysis of target genes via GO and KEGG-based tools. General and cortex-specific protein–protein interaction networks were constructed from the target genes of top miRNAs to assess their functional connections. (4) Conclusions: We demonstrated that plasma microRNA profiles are promising diagnostic and prognostic molecular biomarkers that may find an actual application in the clinical practice of GBM, although more studies are needed to validate our results.

## 1. Introduction

Glioblastoma multiforme (GBM) is one of the most aggressive and lethal primary tumors of the central nervous system accounting for over 80% of malignant gliomas. The prognosis for malignant gliomas has not significantly improved in the last four decades. Fighting the disease is challenging both for patients and health care systems. Despite recent progress that has been made in treatment protocols, GBM is still characterized by a high mortality rate: the average life expectancy of GBM patients (GPs) is 1.5 years [1]. Early detection and assessment of GBM pathologies still need to be solved. Routine diagnostic procedures carried out in clinics are not suitable for early diagnosis and for efficient treatment; therefore, there is a need for new diagnostic and prognostic markers that might also represent novel therapeutic targets [2]. Preferably, these should be detectable in easily accessible biological fluids using robust and minimally invasive methods. During recent years, several groups investigated the possibility of using circulating miRNAs as candidate biomarkers for diagnosis in many human cancers including GBM [3].

MiRNAs are 21–25 nucleotides long, endogenously expressed non-coding RNAs that post-transcriptionally repress the expression of protein-coding genes through binding to the 3′ untranslated regions (UTR) of target mRNAs [4,5]. Accumulated evidence indicates that miRNAs as post-transcriptional regulators may interact with a large number of mRNAs and are involved in the regulation of many biological processes, such as developmental timing, cell metabolism, cell differentiation, cell death, cell proliferation, hematopoiesis, and patterning of the nervous system [6]. Many miRNAs exhibit tissue-specific patterns of expression and are deregulated in various cancers, where they may be either oncogenic (oncomirs) or tumor suppressive. However, this classification is not straightforward: due to an extensive palette of target genes, the same miRNA may play opposing roles in different processes [7]. MiRNAs from tumors may be secreted within membrane vesicles (exosomes) or directly into the blood, indicating that miRNAs probably play a key role in intercellular communication [8,9]. Circulating miRNAs found in human blood plasma may represent stable biomarkers as a result of their packaging into vesicles or interaction with proteins that protect miRNAs from RNase digestion [10].

In addition to analyzing the potential of single miRNAs as biomarkers, there are several research groups that have been investigating the potential of using multiple serum miRNAs in combination for diagnostic and prognostic purposes in GBM. A study revealed that a seven-miRNA panel including hsa-miR-15b, hsa-miR-23a, hsa-miR-150, hsa-miR-197, and hsa-miR-548b-5p had a high potential to distinguish malignant astrocytomas from normal controls [11]. A meta-analysis performed by Qu et al. concluded that panels containing miR-21 may be more specific for glioma [12]. Roth et al. were able to identify a specific miRNA signature in the blood cells of GPs, namely an increased expression of hsa-miR-128 and hsa-miR-194 and a decreased expression of hsa-miR-342-3p and hsa-miR-628-3p [13]. Despite a large number of reports, no consensus regarding the circulating miRNA signature has been reached so far, making it currently impossible to unambiguously distinguish GBM patients from healthy individuals (HIs) on this basis.

NCounter technology may be used to detect any type of nucleic acid in solution and—with modifications—to assess other biological molecules as well. In this study, we chose this assay for profiling miRNA expression in plasma samples of GPs and HIs. Differentially expressed (DE) miRNAs were identified, their targets were predicted by a bioinformatics search and subjected to gene ontology and pathway analyses. Detection of the significantly differently expressed miRNAs in plasma samples of more GPs could provide valuable insight into the pathogenesis of GBM.

## 2. Results

### 2.1. Identification of Differently Expressed MicroRNAs in Plasma Samples of GBM Patients and Healthy Controls

In order to compare plasma miRNA expression profiles between GPs and HIs, six GBM patients and six individuals diagnosed with disc herniation—serving as healthy controls—were recruited in this study. Patients were aged between 52 and 69 years with a mean age of 61.3 years, while the age of controls ranged from 42 to 68 years with a mean age of 58.6 years. In both groups, the number of women and men were equal.

MiRNA profiles in plasma samples were determined using the nCounter Human v3 miRNA Panel of the NanoString nCounter Analysis System (NanoString Technologies, Seattle, WA, USA). During the experiment, special attention was paid to sampling and sample processing in order to preserve miRNA stability and enable standardization of the results.

Normalized miRNA counts varied considerably among individuals; however, most miRNA counts were low, especially in controls, with few exceptions. Hsa-miR-451a had the highest count with group mean values 1144 and 2965 for controls and GBM patients, respectively. It was followed by hsa-let-7i-5p and hsa-miR-6721-5p (group mean values: 233 and 349; 179 and 254, respectively). None of these highly expressed miRNAs showed significantly different expression among the cohorts.

**Figure 1:**
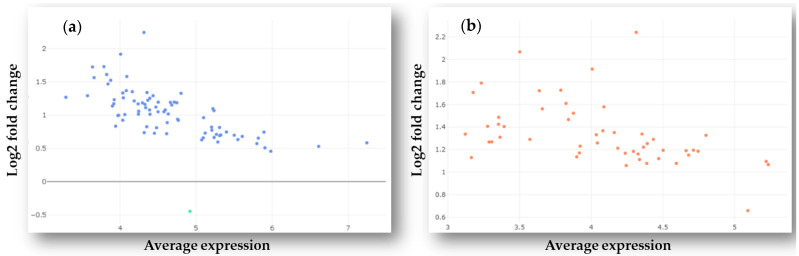
MA plots showing the log_2_ fold change of normalized counts in the function of the average expression values of differentially expressed miRNAs in plasma samples of GBM patients. (**a**) Expression data were filtered for false discovery rate (FDR) 0.15 and fold change 0.5; (**b**) The same data were filtered for FDR 0.1 and fold change 1.

In total, 107 out of the 798 unique miRNAs showed significant differences in counts between tumor and normal plasma samples following filtering and differential expression analysis. After adjusting expression data for fold change (FC) and false discovery rate (FDR) cut-off values (log_2_FC 1 and FDR 0.1), 52 miRNAs were found to be upregulated, among them hsa-miR-338-5p had the highest expression value (log_2_FC = 2.14). Hsa-miR-181a-3p was the only downregulated miRNA in GP samples compared to healthy controls (log_2_FC = −0.43) after adjusting for FDR 0.1. The distribution of DE miRNAs is shown in Figure 1a,b. The complete list of DE miRNAs is presented in Table 1. Based on our search results using the miRCancer and miR2Disease databases and the literature, the majority (~94%) of the DE miRNAs identified in this study had already been found to be associated with various malignancies, many of them (~57%) with GBM, too.

### 2.2. Validation of Differentially Expressed MiRNAs by RT-qPCR

To validate our NanoString results, five miRNAs (hsa-miR-433-3p, hsa-miR-362-3p, hsa-miR-195-5p, hsa-miR-133a-3p, and hsa-miR-29a-3p) were randomly chosen together with hsa-miR-1286-3p, which did not show differential expression between the two groups. Relative expression of these miRNAs was determined by RT-qPCR measurements using hsa-miR-16-5p as the reference miRNA [14,15]. All measurements were done in triplicate. Expression of hsa-miR-433-3p, hsa-miR-195-5p, and hsa-miR-29a-3p was significantly upregulated compared with those in the control samples (the Kruskal–Wallis *p*-values were the following: hsa-miR-433-3p: *p* = 0.00714, hsa-miR-195-5p: *p* = 0.0466, hsa-miR-29a-3p: *p* = 0.0041), while in the case of hsa-miR-362-3p and hsa-miR-133a-3p we could not detect any expression either in healthy control samples or in GBM patient samples. Although RT-qPCR methods for miRNA quantification are relatively inexpensive, commonly available, and allow measurements of very small quantities of miRNAs, the amounts of circulating miRNAs in peripheral blood are often below their limit of detection [15]. Expression of hsa-miR-1286-3p did not show a significant difference between HIs and GPs.

**Figure 2:**
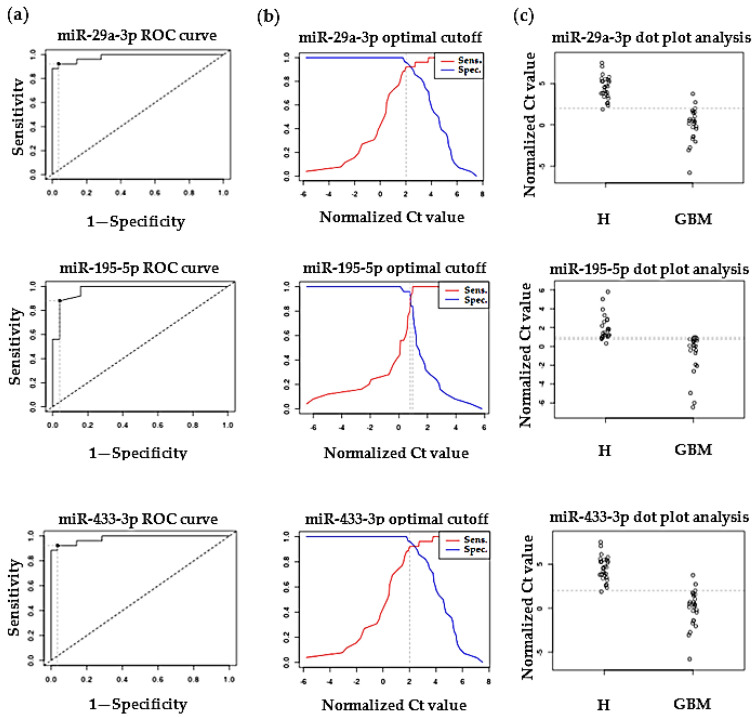
(**a**) ROC (receiver operating characteristics) curves with AUC (area under the curve) were made to check and visualize the performance of miR-29a-3p, miR-195-5p, and miR-433-3p, randomly selected for the validation of the NanoString measurement via qRT-PCR. (**b**) Estimated optimal cut-point values of miR-29a-3p (normalized Ct value: 2), miR-195-5p (normalized Ct value: 0.97), and miR-433-3p (normalized Ct value: 2). (**c**) Dot plot analysis of miR-29a-3p, miR-195-5p, and miR-433-3p for GBM patients (GBM) and healthy controls (H).

We constructed ROC-AUC curves using the expression data obtained from the non-malignant and malignant samples for hsa-miR-433-3p, hsa-miR-195-5p, and hsa-miR-29a-3p that showed significantly different expression between HIs and GPs according to NanoString and RT-qPCR measurements. ROC-AUC proved to be 0.98214, 0.9704, and 0.98214 in the case of hsa-miR-433-3p, hsa-miR-195-5p, and hsa-miR-29a-3p, respectively. Normalized Ct values for HIs and GPs were dichotomized by mapping the sensitivity values in relation to 1—specificity in the case of hsa-miR-433-3p, hsa-miR-195-5p, and hsa-miR-29a-3p—to estimate optimal cut-off values. Hsa-miR-433-3p and hsa-miR-29a-3p showed the same sensitivity (92%) and specificity (96%), while in the case of hsa-miR-195-5p sensitivity was 88% and specificity was 96% (Figure 2). Of course, these results require validation in a larger cohort of GBM patients.

### 2.3. MiRNA Ranking, Target Gene Prediction, and Analysis

An accurate prediction of miRNA targets is critical for characterizing the function of DE miRNAs. The facts that a single miRNA may interact with several different mRNAs and the translation of an mRNA may be regulated by several miRNAs justify a network-based analysis for miRNA function. In this study, the miRNet tool was applied to identify experimentally validated miRNA targets and build an miRNA-centric network incorporating direct miRNA–target gene interactions and protein–protein interactions into the network. This approach allows us to determine the importance of the given miRNAs in the network based on their network-specific centrality values. On the basis of this analysis, hsa-miR-215-5p has the highest degree value (755) followed by hsa-miR-195-5p (640) and hsa-miR-362-3p (536), reflecting their importance in the network. (Complete and minimum networks built from the DE miRNA–target gene and protein–protein interactome of proteins encoded by target genes—as generated by the miRNet tool—are presented in Supplementary Materials Figure S1).

**Figure 3:**
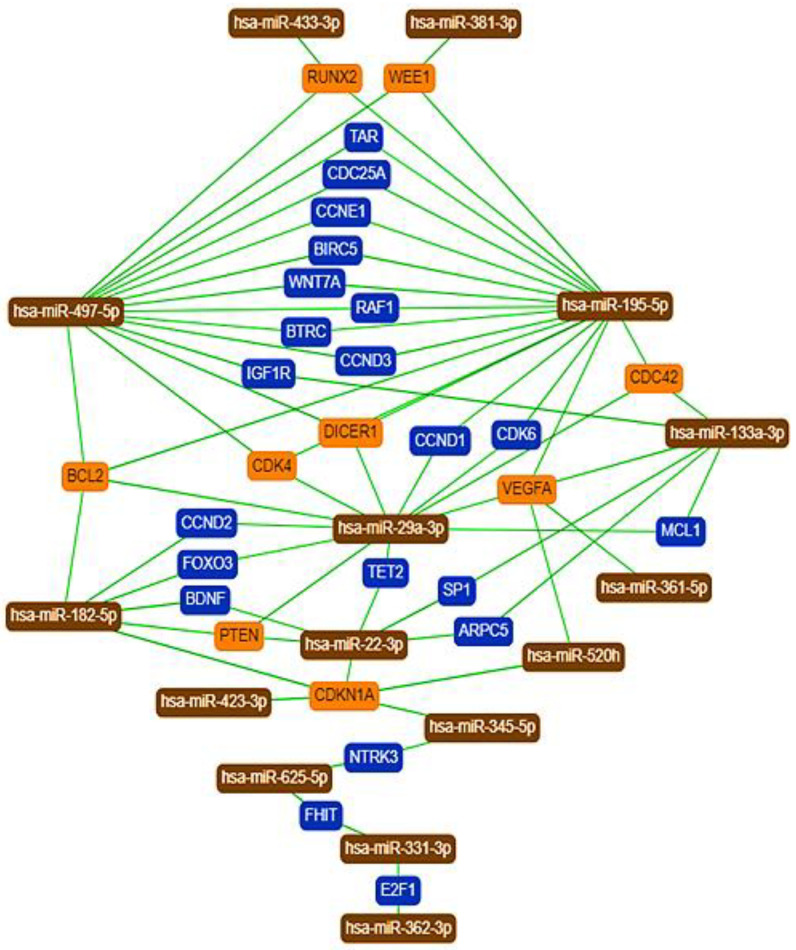
The core network of DE miRNAs and their experimentally validated target genes. The network was generated by the miRTargetLink tool using the strong interaction option. Color code: orange, more than two interactions; blue, two interactions in the full network.

Applying the miRTargetLink tool, we constructed a core miRNA–target network to show the experimentally validated miRNA–target interactions for the top 10 miRNAs with the highest expression values from the significantly DE miRNAs revealed by our analysis. We also included hsa-miR-433-3p, hsa-miR-362-3p, hsa-miR-195-5p, hsa-miR-133a-3p, and hsa-miR-29a-3p: miRNAs used for the RT-qPCR validation of the NanoString measurement (Figure 3). According to the miRTargetLink tool, their common target genes possessing more than two interactions in the full network are Runt-related transcription factor 2 (RUNX2), nuclear kinase (WEE1, a key regulator of cell cycle progression), cell division control protein 42 homolog (CDC42), B-cell lymphoma 2 (BCL2, a regulator of apoptosis), cyclin-dependent kinase 4 (CDK4), endoribonuclease (DICER1), vascular endothelial growth factor A (VEGFA), phosphatase and tensin homolog (PTEN), and cyclin-dependent kinase inhibitor 1A (CDK1NA). All of these proteins are known to be involved in tumorigenesis (Figure 3) [16,17,18,19].

### 2.4. Pathway and Gene Ontology Enrichment Analysis of MiRNA Targets

**Figure 4:**
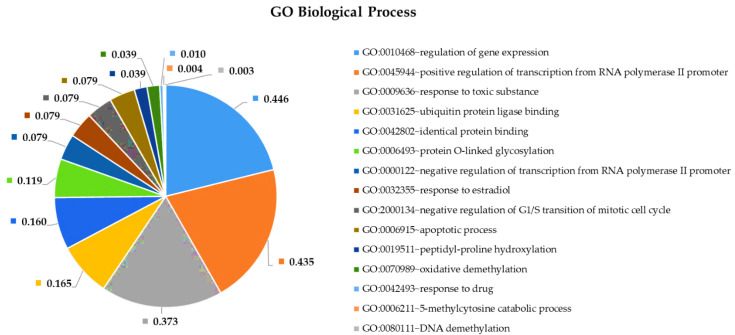
Functional annotation enrichment of target genes of the top 38 deregulated miRNAs based on their enrichment in Gene Ontology GO_ Biological Processes. Significance of the selected (?) pathways are characterized by their FDR values shown inside the chart sections.

**Figure 5:**
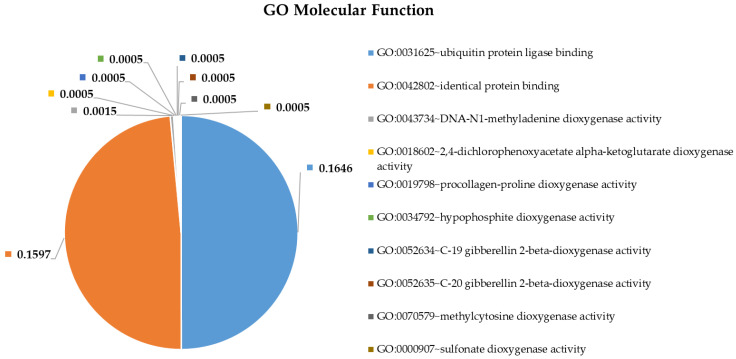
Functional annotation enrichment of target genes of the top 38 deregulated miRNAs based on their enrichment in Gene Ontology GO_ Molecular Function. Significance of the selected (?) pathways are characterized by their FDR values shown inside the chart sections.

**Figure 6:**
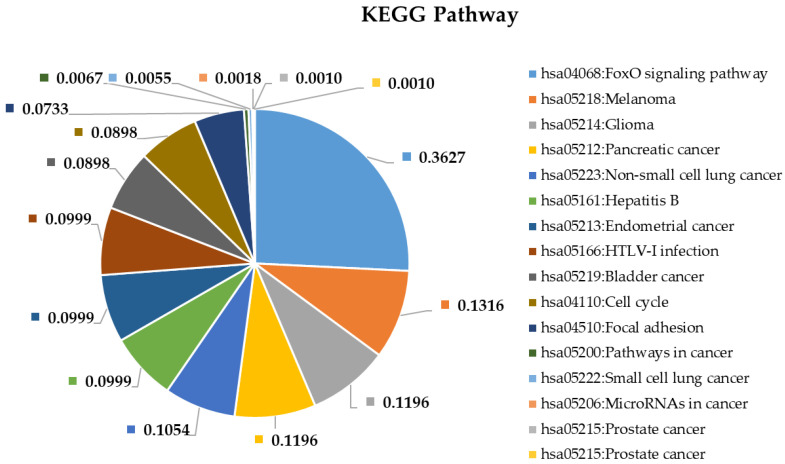
Functional annotation enrichment of target genes of the 38 most deregulated miRNAs based on their enrichment in specific Kyoto Encyclopedia of Genes and Genomes (KEGG) pathways. Significance of the selected (?) pathways are characterized by their FDR values shown inside the chart sections.

To shed more light on the potential pathophysiological role of significantly DE miRNAs in GBM development and to further explore the function of their predicted target genes, we selected the most upregulated 38 miRNAs and the single downregulated hsa-miR-181a-3p to perform Gene Ontology and pathway enrichment analysis. A functional annotation and enrichment analysis of their target genes in Gene Ontology Biological Process (GO-BP), Molecular Function (GO-MF) terms, and in canonical KEGG pathways was performed using the DAVID tool [20]. Among the identified biological processes, we found DNA demethylation, protein O-linked glycosylation, positive regulation of transcription from RNA polymerase II promoter, negative regulation of transcription from RNA polymerase II promoter, regulation of gene expression, negative regulation of G1/S transition of the mitotic cell cycle, and the apoptotic process. The complete list of GO-BP and GO-MF for the 39 DE miRNAs is present in Figure 4 and Figure 5. Furthermore, as far as can be judged from the result of a KEGG pathway analysis, a number of cancer types such as glioma, prostate cancer, bladder cancer, small cell lung cancer, non-small-cell lung cancer, melanoma, endometrial cancer, pancreatic cancer, and viral infectious pathways (hepatitis B, HTLV-I infection) were found to be associated (Figure 6).

### 2.5. Protein–Protein Interaction Network Analysis of MiRNA Targets

**Figure 7:**
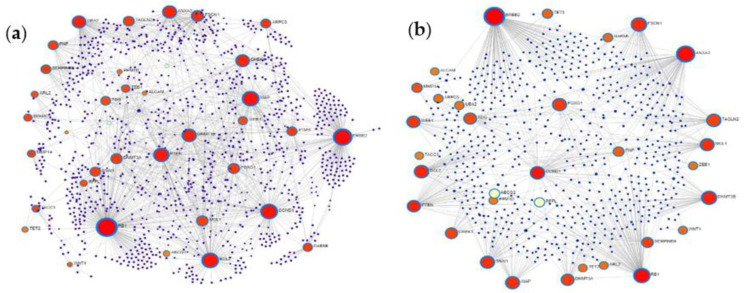
Topology of the general and cortex-specific protein–protein interaction (PPI) minimum networks constructed from common targets of DE miRNAs using the NetworkAnalyst tool. Part (**a**,**b**): The general and cortex-specific minimum PPI networks, respectively. Nodes represent proteins, only the major hub nodes are labeled in the networks. The size of the nodes corresponds to their degree centrality.

Based on common protein targets of the 38 most upregulated miRNAs and the only downregulated miRNA, we constructed two protein–protein interaction (PPI) networks. First, a general PPI network, then a cortex-specific PPI network was generated from the target list by using the NetworkAnalyst tool. Both of them proved to be large fuzzy networks: there are 1191 nodes and 1676 edges in the general PPI network, while the cortex-specific one contains 668 nodes and 852 edges. In Figure 7, the general and cortex-specific minimum networks are shown with the major hubs labeled.

Comparing the two networks, it can be established that many of the major hubs—considered to be key nodes with major biological importance—are shared and represent proteins that are already known to be involved in tumorigenesis, such as BCL2, retinoblastoma 1 (RB1), PTEN, erb-b2 receptor tyrosine kinase 2 (ERBB2), cyclin D1 (CCND1), zinc finger E-box binding homeobox 1 (ZEB1), fascin actin-bundling protein 1 (FSCN1), Wnt family member 1 (WNT1), X-linked inhibitor of apoptosis (XIAP), forkhead box O1 (FOXO1), ubiquitin like modifier activating enzyme 2 (UBA2), DNA methyltransferase 3 beta (DNMT3B), annexin A2 (ANXA2), and WEE1. However, ranking of nodes based on their degree centrality is not identical in the two PPI networks. ERBB2, RB1, ANXA2, CCND1, PTEN, FSCN1, DNMT3B, BCL2, XIAP, FOXO, and WEE1 are present in both but have higher degree centrality in the cortex-specific network.

**Figure 8:**
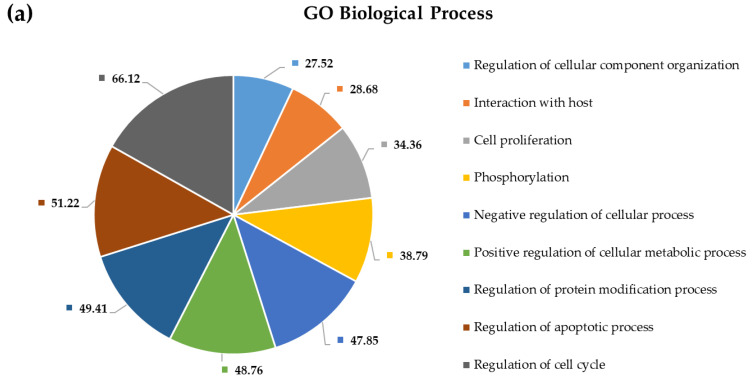

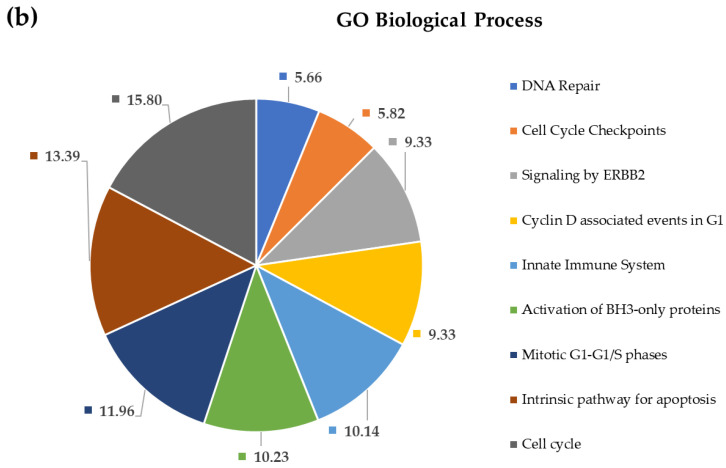
GO biological process–based general (**a**) and cortex-specific (**b**) functional enrichment annotation of all target genes of differentially expressed miRNAs applying the NetworkAnalyst tool. Significance of the selected (?) pathways are characterized by their FDR values shown inside the chart sections.

**Figure 9:**
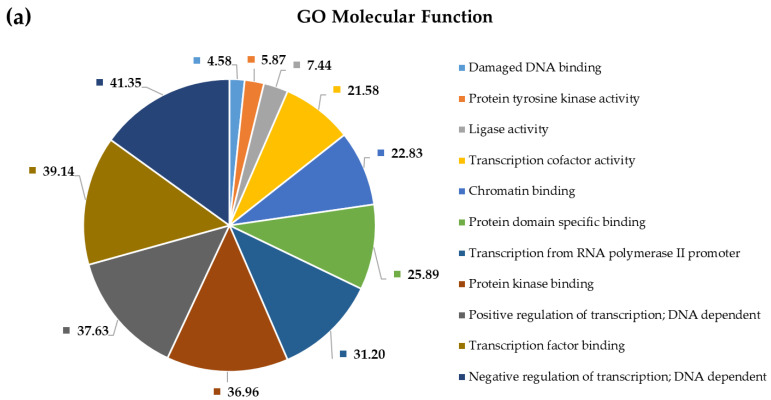

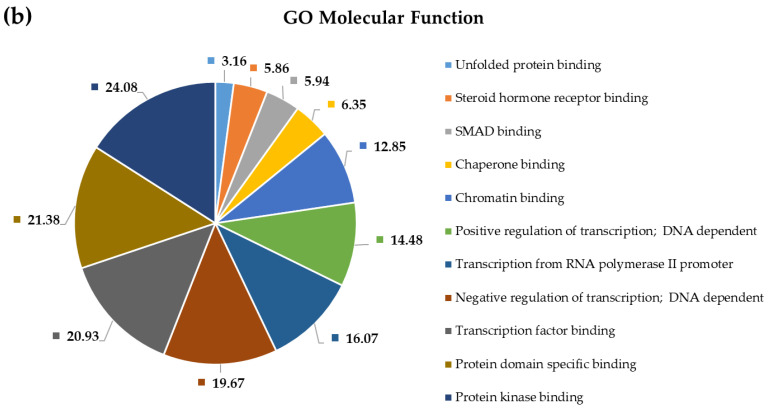
GO molecular function–based general (**a**) and cortex-specific (**b**) functional enrichment annotation of all target genes of differentially expressed miRNAs applying the NetworkAnalyst tool. Significance of the selected (?) pathways are characterized by their FDR values shown inside the chart sections.

**Figure 10:**
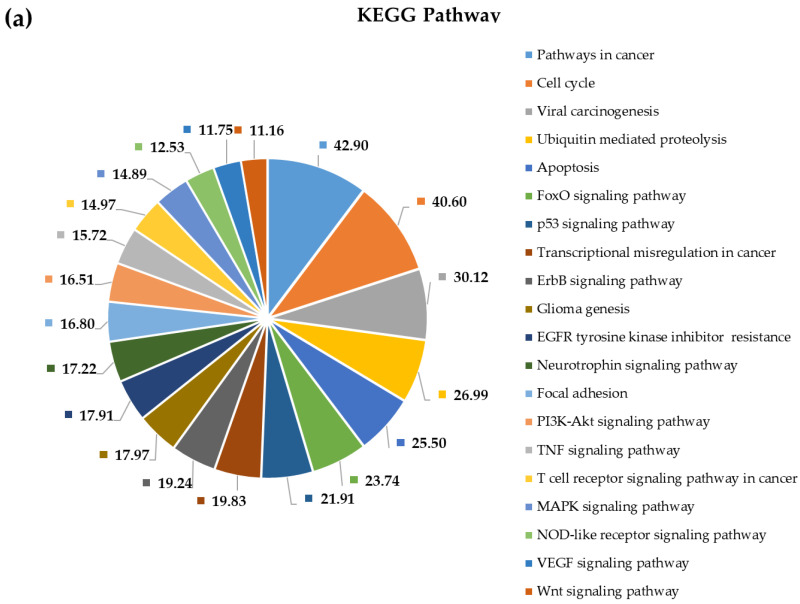

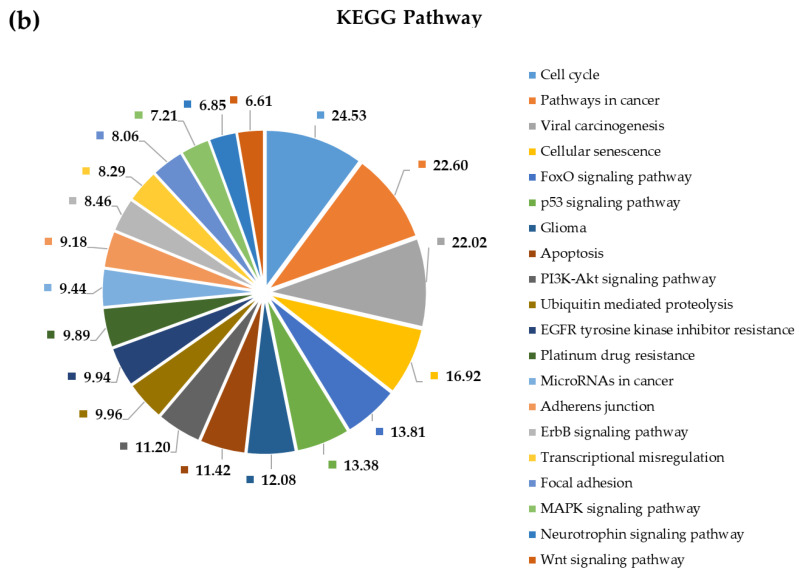
KEGG pathway–based general (**a**) and cortex-specific (**b**) functional pathway enrichment annotation of all target genes of differentially expressed miRNAs applying the NetworkAnalyst tool. Significance of the selected (?) pathways are characterized by their FDR values shown inside the chart sections.

Using the Gene Ontology and KEGG database options of the NetworkAnalyst tool, we performed a PPI-network-based functional enrichment and pathway analysis as well. This made it possible to compare results of the general analysis with results of a cortex-specific enrichment analysis. Results are shown in Figure 8 and Figure 9. Results of the functional enrichment analysis suggest that circulating plasma miRNAs are not randomly released from cells, since many of their predicted target genes are enriched in critically important pathways and biological processes contributing to tumorigenesis. Examples for that are pathways in cancer, cell cycle, viral carcinogenesis, ubiquitin-mediated proteolysis, apoptosis, FoxO signaling pathway, p53 signaling pathway, glioma genesis, transcriptional misregulation in cancer, ErbB signaling pathway, EGFR tyrosine kinase inhibitor resistance, PI3K–Akt signaling pathway, and neurotrophin signaling pathway. Functional enrichment analysis of target genes using the KEGG pathways also revealed several cancer types; however, those are not listed in Figure 10. Mazurek et al. analyzed the molecular pathways of tumor metabolism in GBM. According to their analysis, the activation of PI3K results in the formation of phosphatidylinositol-3,4,5,-triphosphate (PIP3), and following its formation, PtdIns(3,4,5)P3 recruits Akt at the inner plasma membrane. This translocation of Akt into the membrane activates its kinase activity, leading to uncontrolled cell proliferation and the inhibition of apoptosis during tumor transformation. Growth factor receptors are other elements of the PI3K axis and are involved in its activation. These include EGFR, EGFRvIII, and platelet-derived growth factor receptors (PDGFRs) [21].

## 3. Discussion

Glioblastoma (GBM) is a devastating primary malignancy of the central nervous system. Fighting the disease is challenging both for patients and health care systems. Survival is poor, and treatment options are limited [22]. In addition to imaging studies, biomarkers—especially ones detectable from liquid biopsy—may be helpful to distinguish between healthy persons and GPs, to detect tumor recurrence at the earliest possible stage, and to distinguish between pseudoprogression and substantial tumor growth [23].

Data regarding the composition of the human genome show that a large part of it is transcribed into non-coding RNAs with major functions both in normal physiology and in pathological processes [24]. In recent years, several groups examined the biological importance of cell-free miRNAs present in body fluids. It was suggested that circulating miRNAs have the potential to become non-invasive biomarkers for the early diagnosis of cancer [25,26]. MiRNAs have the advantage of being clearly defined markers that may easily be determined by microarrays, nCounter technology, or real-time PCR in peripheral blood. These methods allow the analysis of specific miRNA patterns that comprise numerous miRNAs.

### 3.1. Identification of Differently Expressed MiRNAs in Plasma Samples of GBM Patients and Healthy Controls

#### 3.1.1. NanoString Analysis and Identification of Differentially Expressed MiRNAs

We performed miRNA screening analyses of plasma samples of GBM patients and healthy individuals using nCounter. We compared expression profiles of circulating miRNA in blood plasma samples of six healthy persons and six GBM patients. The nCounter Human v3 miRNA Panel of the NanoString System was used to measure miRNA levels. MiRNA counts were low for most of the miRNAs, especially in the control samples. This may be due to the detection method: NanoString does not require an amplification step, so it is clearly different from methods that use PCR for miRNA measurement.

Comparing miRNA expression profiles in the control and GBM patient samples, we have identified 59 miRNAs showing significantly different expression between controls and patients: 58 miRNAs were found to be upregulated, whereas 1 was downregulated. The observed significantly deregulated 53 miRNAs amounted to 6.64% of the 798 miRNAs analyzed in total. The majority (~93%) of differentially expressed miRNAs identified in this study had been previously associated with various malignancies, many of them (~57%) with GBM too [27,28]. On the basis of the NanoString measurement, the only downregulated miRNA in GPs was hsa-miR-181a-3p (log_2_FC = −0.43), whereas the most strongly upregulated one was hsa-miR-338-5p (log_2_FC = 2.14). Very recently, He et al. performed a meta-analysis of 24 studies comprising 2170 patients with different grades of glioma and 1456 healthy participants to evaluate the diagnostic potential of circulating miRNAs. Overall, 6 miRNAs (hsa-miR-133, hsa-miR-181, hsa-miR-182, hsa-miR-197, hsa-miR-497, and hsa-miR-548) were noted as significantly differentially expressed by both our own study and by He et al. [29].

#### 3.1.2. Differently Expressed MiRNAs Specific for GBM

Searching the miR2Disease and miRCancer databases, we found that out of our pool of significantly deregulated miRNAs, two miRNAs (hsa-miR-1252-5p and hsa-miR-591) showed new associations with GBM. Rodrigues-Junior et al. revealed a direct association between bortezomib sensitivity in multiple myeloma cells by targeting heparanase (HPSE). Moreover, overexpression of miR-1252-5p significantly reduced HPSE expression and HPSE enzymatic activity in MM cells [30]. In addition, there are several KEGG pathways related to tumorigenesis signaling identified as putative targets for miR-1252-5p. These pathways are linked to lysine degradation, transcriptional misregulation, glycosphingolipid, and proteoglycan biosynthesis [30]. Huh et al. demonstrated that the regulation of miR-106a and miR-591 in ovarian cancer cells affects sensitivity to paclitaxel (PTX) and cancer cell migration and proliferation, and that ZEB1, BCL10, and caspase-7 are direct target genes of miR-106a and miR-591 [31]. Hsa-miR-423-3p should also be noted as specific for GBM and having an experimentally validated target, CDKN1A, which is a component of pathways known to play key roles in GBM biology [32].

#### 3.1.3. The Role of Hsa-miR-433-3p in Cancer Development

Dysregulated hsa-miR 433-3p expression has been observed in various cancers and has been significantly associated with the clinical outcome of tumor patients. It was reported that hsa-miR-433-3p was downregulated in gastric carcinoma [33], visceral adipose tissue of patients with non-alcoholic steatohepatitis [34], and hepatitis B virus–associated hepatocellular carcinoma (HCC) [35]. Previous studies reported that hsa-miR-433-3p was downregulated in glioblastoma samples as well [36,37]. For example, Sun et al. revealed that hsa-miR-433-3p is downregulated in glioma tissue and cells and functions as a tumor suppressor by targeting CREB in glioma, thus regulating cell growth, invasion, and migration [36]. As shown in the present study for GBM, hsa-miR-433-3p was reported to be upregulated in patient plasma samples compared to control plasma samples in human osteosarcoma. In osteosarcoma, ectopic expression of hsa-miR-433 decreased apoptosis in tumor cells by targeting programmed cell death 4 (PDCD4), indicating that hsa-miR-433 may be a potential molecular target for osteosarcoma treatment [38]. To further confirm the upregulation of hsa-miR-433-3p in GBM, RT-qPCR analysis was performed. Hsa-miR-433-3p expression was markedly higher in GP plasma samples than in healthy control samples. Given the differences in sample sources, RNA extraction, and detection methods, more studies are required to further assess the role of hsa-miR-433-3p in GBM.

### 3.2. Validation of Differentially Expressed MiRNAs by RT-qPCR

In addition to hsa-miR-433-3p, the relative expression of some other miRNAs was also determined by RT-qPCR measurements using hsa-miR-16-5p as the reference miRNA. Expression of hsa-miR-195-5p and hsa-miR-29a-3p was significantly upregulated compared with those in the control samples, while in the case of hsa-miR-362-3p and hsa-miR-133a-3p we could not detect any expression either in healthy control samples or in GBM patient samples. Similarly to our results, Wang et al. found the upregulation of hsa-miR-195-5p in both blood and tissues of GBM patients and suggested that it may regulate fatty acid metabolism through predicted or validated target genes [39]. It was found that patients with high-risk/relapsed tumors are intimately linked to metabolic abnormalities, e.g., fatty acid biosynthesis has been reported to play important roles in the pathogenesis of multiple cancers [40]. Jia et al. observed that median survival of patients with low hsa-miR-195 levels was 15 months, whereas for patients with high hsa-miR-195 levels, it was 56.53 months. Multi-factor Cox regression analysis showed that a high level of hsa-miR-195 (odds ratio (OR): 0.347, 95% CI: 0.121–0.992) was associated with decreased mortality of patients [41]. Zhao et al. found that hsa-miR-29a downregulates PTEN, EphB3, and SOX4 expression to activate a complex post-transcriptional program of growth and invasion in glioblastoma that promotes glioblastoma aggressiveness. In addition, increased hsa-miR-29a expression in glioblastoma specimens correlates with decreased patient survival [17]. About hsa-miR-181a-3p, which we detected using NanoString was the only downregulated miRNA, Shi et al. found that hsa-miR-181a and hsa-miR-181b function as tumor suppressors triggering growth inhibition, inducing apoptosis, and inhibiting invasion in glioma cells. These findings suggest aberrantly downregulated hsa-miR-181a and hsa-miR-181b may be critical factors that contribute to malignant appearance in human gliomas [42].

### 3.3. MiRNA Ranking, Target Gene Prediction, and Analysis

Using the miRNet tool and a network-based approach, we constructed a miRNA–target interaction network for miRNA groups. MiRNAs were ranked based on their degree-centrality value in the network, which reflects their biological importance. Applying the mirTargetLink tool, we constructed core miRNA–target networks to show the strongest interactions among 15 DE miRNAs including the ones we used for RT-qPCR analysis. According to mirTargetLink, their common target genes showing more than two interactions in the full network are RUNX2, WEE1, CDC42, BCL2, CDK4, DICER1, VEGFA, PTEN, and finally CDK1NA. Yamada et al. detected RUNX2 protein in five out of seven human GBM cell lines, and its level was positively correlated with proliferative capacity [43]. Wu et al. concluded that a combinational inhibition of WEE1 and PI3K might allow successful targeted therapy in GBM [44]. According to Okura et al., high Cdc42 expression is associated with poorer progression-free survival, and Cdc42 expression is highest in the proneural and neural subgroups of GBM [16]; similarly, the expression of *bcl-2* had a significant relationship with survival as well [19]. Li et al. revealed that proneural GBM has increased vulnerability to CDK4/6 inhibition, and the proneural subtype undergoes dynamic reprogramming upon palbociclib treatment, suggesting the need for a combination therapy [45]. Findings by Johansson et al. suggest that elevated expression of VEGF-A may be a prerequisite for the aggressive and infiltrative growth of astrocytomas [46]. Several reports have shown that PTEN may control tumorigenesis independent of its enzymatic activity, through its interaction with specific nuclear proteins. Benitez et al. demonstrated that PTEN regulates glioblastoma oncogenesis through chromatin-associated complexes of DAXX and histone H3.3 [47].

### 3.4. Pathway and Gene Ontology Enrichment Analysis of MiRNA Targets

MiRNA-group-specific target lists were used in a functional annotation analysis based on the enrichment of miRNA targets in KEGG pathways and terms of gene ontology biological processes. This revealed that miRNA targets are enriched in known cancer pathways: signaling pathways crucial to tumorigenesis. Among the identified biological processes, we find DNA demethylation, protein O-linked glycosylation, positive regulation of transcription from RNA polymerase II promoter, negative regulation of transcription from RNA polymerase II promoter, regulation of gene expression, negative regulation of G1/S transition of the mitotic cell cycle, and the apoptotic process. As far as can be judged from the results of a KEGG pathway analysis, several cancer types such as glioma, prostate cancer, bladder cancer, small cell lung cancer, non-small-cell lung cancer, melanoma, endometrial cancer, pancreatic cancer, and viral infectious pathways (hepatitis B, HTLV-I infection) were shown to be associated.

### 3.5. Protein–Protein Interaction Network Analysis of MiRNA Targets

Possible interactions between target proteins and their functionally important interacting protein partners were analyzed by constructing general and cortex-specific PPI networks. The major hub proteins—BCL2, RB1, PTEN, ERBB2, CCND1, ZEB1, FSCN1, WNT1, XIAP, FOXO1, UBA2, DNMT3B, ANXA2, and WEE1—were basically the same in the two networks, suggesting that our differentially expressed miRNAs regulate target genes that are involved in basic processes of tumor formation. Applying the NetworkAnalyst tool, we performed a network-based functional and pathway enrichment analysis as well, including pathways in cancer, cell cycle, viral carcinogenesis, ubiquitin-mediated proteolysis, apoptosis, FoxO signaling pathway, p53 signaling pathway, glioma genesis, transcriptional misregulation in cancer, ErbB signaling pathway, EGFR tyrosine kinase inhibitor resistance, PI3K-Akt signaling pathway, and neurotrophin signaling pathway, to name a few of the most relevant ones. Key GO-BP terms included regulation of cell cycle, regulation of apoptotic process, regulation of protein modification process, positive regulation of cellular metabolic process, and negative regulation of cellular process, while among GO-MF terms, enzyme binding, negative regulation of transcription, kinase binding, transcription factor binding, positive regulation of transcription, and chromatin binding were the most crucial ones based on target enrichment and over-representation. Results of enrichment analysis show that most miRNA targets are involved in signaling pathways and biological processes critical for tumor formation, suggesting that circulating miRNAs could be potential regulatory factors in tumorigenesis. At the same time, these data also show that the identified enriched pathways and GO terms are not specific for a given tumor type. A network-based approach could provide a means to discover novel proteins, which interact physically and functionally with the seed proteins and may represent new cancer genes or cancer biomarkers.

## 4. Materials and Methods

### 4.1. Patients and Samples

Glioblastoma (GBM) patients (GPs) were detected and treated at the Department of Neurosurgery, Faculty of Medicine, University of Debrecen, Hungary. Blood samples were collected from six individuals diagnosed with disc herniation, serving as healthy controls (HIs), and six patients with a histopathological diagnosis of GBM (GPs). Written informed consent was obtained from each study subject. Demographic and clinical data for the study subjects were obtained from the medical record review. None of the patients received chemotherapy or radiotherapy treatment prior to participation in this study. Preoperative blood samples were taken at admission or at the time of anesthesia induction for tumor resection. The study was approved by the Scientific and Research Ethics Committee of the Medical Research Council of the Ministry of Health, Budapest, Hungary (ETT TUKEB; project identification code: 51450/2015/EKU (0411/15)) and was conducted in accordance with the Declaration of Helsinki.

Whole-blood samples were collected in EDTA anticoagulated tubes (BD Vacutainer, Euromedic, Budapest, Hungary) from each patient and from healthy volunteers for plasma isolation and kept at 4 °C until further processing (within two hours of collection). Whole blood was subjected to a two-step centrifugation protocol (2500× *g* and 16,000× *g*; 10–10 min, 4 °C) to obtain plasma. After separation, cell-free plasma samples were homogenized, aliquoted, and stored at −80 °C until further processing.

### 4.2. RNA Isolation and Purification for NanoString Measurement

Plasma samples were thawed at room temperature and total cell-free RNA was extracted from 500 μL of plasma and purified using the miRNeasy Serum/Plasma RNA isolation kit (Qiagen, Hilden, Germany) according to the manufacturer’s instructions. The quality of RNA was checked using a Nanodrop device (Thermo Scientific, Waltham, MA, USA).

### 4.3. NanoString Analysis and Identification of Differentially Expressed MiRNAs

MiRNA content of all plasma samples was analyzed using the nCounter Human v3 miRNA Panel of NanoString nCounter Analysis System (NanoString Technologies, Seattle, WA, USA) with 798 unique hsa-miRNA barcodes on it. Digital nCounter miRNA profiling technology is capable of accurately discriminating between miRNAs at a single-base resolution in a complex mixture. The system provides a direct digital readout of each miRNA without requiring cDNA synthesis or enzymatic reactions. For the analysis, 100 ng total cell-free RNA was used from each sample and mixed with pairs of capture and reporter probes tailored to specifically recognize each miRNA present. Overnight hybridization (16 to 20 h) at 65 °C allowed sequence-specific probes to form complexes with targets. Excess probes were removed using two-step magnetic-beads-based purification on an automated fluidic handling system (nCounter Prep Station, Thermo Scientific, Waltham, MA, USA), and target–probe complexes were immobilized on the cartridge for data collection. Data collection was carried out on the nCounter Digital Analyzer (NanoString Technologies, Seattle, WA, USA) following the manufacturer’s instructions, to count individual fluorescent barcodes and quantify target RNA molecules present in each sample. For each assay, a high-density scan (600 fields of view) was performed.

Background correction of data was performed by subtracting the mean ± 2 standard deviation of the negative control set. Lane-by-lane technical variation was corrected by using the geometric median value of the positive-control set. Following correction, an independent prefiltering step was performed to remove miRNAs having less than three counts in any sample. A nonparametric Mann–Whitney U test was used to identify significantly DE miRNAs between the two groups. The raw count matrix of DE miRNAs was used to determine quantitative changes in expression levels between glioblastoma patients and the control group. Analysis of miRNA expression data was carried out by the iDEP tool, a Shiny app powered by several R and Bioconductor packages [48]. For differential expression analysis, the DESeq2 package was used. After adjusting the cut-off values for fold change (FC ≥ 1) and false discovery rate (0.1), 52 upregulated DE miRNAs were identified. A single downregulated miRNA was identified by lowering the FC cut-off value.

### 4.4. Prediction of Targets of Differentially Expressed MiRNA, Functional Annotation, and Pathway Enrichment Analysis

First, a miRNA–target gene network was constructed using the web-based miRNet tool (http://www.mirnet.ca, accessed on 2 February 2021). Top miRNAs in the network were ranked by degree- and betweenness-centrality values. Prediction of experimentally validated target genes of miRNAs was carried out using the web-based miRNet, miRTarBase, and TargetScan software (http://miRTarBase.mbc.nctu.edu; www.targetscan.org, accessed on 8 February 2021). Target intersections were further validated by the miRWalk2 database (http://zmf.umm.uni-heidelberg.deg, accessed on 10 February 2021). General and GBM specific protein–protein interaction (PPI) networks of target genes were constructed using the NetworkAnalyst 3.0 tool (www.NetworkAnalyst.ca, accessed on 12 February 2021).

Lists of miRNA targets were used as input and the online Database for Annotation, Visualization, and Integrated Discovery (DAVID; https://david.ncifcerf.gov, accessed on 13 February 2021) software tool was used to perform gene ontology (GO) and Kyoto Encyclopedia of Genes and Genomes (KEGG)-based functional pathway enrichment analysis for the predicted target genes of prioritized DE hsa-miRNAs.

The NetworkAnalyst tool was used to carry out GBM-specific enrichment analysis. A *p* value of <0.05 was considered statistically significant.

### 4.5. Validation of Hsa-miRNA Expression by Quantitative Real-Time PCR (RT-qPCR)

Total cell-free RNA was extracted from 200 µL plasma samples of 28 healthy control persons and 26 GBM patients by using the miRNeasy Serum/Plasma Kit (Qiagen, Hilden, Germany) including 3.5 µL miRNeasy Serum/Plasma Spike-In Control RNA, according to the manufacturer’s instructions. Concentration of the miRNA fraction in purified total RNA in each sample was measured applying a miRNA-specific fluorometric assay on a Qubit^®^ 2.0 Fluorimeter (Thermo Fischer Scientific, Waltham, MA, USA). To detect and measure the amounts of mature miRNAs, the miScript PCR System (Qiagen, Hilden, Germany) was used. The miScript II RT Kit (Qiagen, Hilden, Germany) was used for reverse transcription of miRNAs. Quantitative real-time PCR was performed on the LightCycler^®^ 96; Roche Molecular Systems (Pleasanton, CA, USA) using the miScript SYBR Green PCR Kit (Qiagen, Hilden, Germany) to determine the expression level of hsa-miR-433-3p, hsa-miR-29-3p, hsa-miR-195-5p, hsa-miR-362-3p, hsa-miR-133a-3p, and hsa-miR-1286-3p in both GBM patient and control samples. Conditions for the PCR reactions were as follows: denaturation at 95 °C for 15 min, followed by 50 amplification cycles of 94 °C for 15 s, 55 °C for 30 s, and 70 °C for 30 s. Finally, a melting curve was generated by taking fluorescent measurements every 0.2 °C for 25 s from 50 °C up to 95 °C to detect a single PCR product. Expression levels of the selected miRNAs were calculated using comparative cycle threshold (Ct) method, and miR-16 was selected as internal control. Fold change in miRNA expression was calculated according to the ΔΔCt method. Experiments were performed in triplicate.

### 4.6. Statistical Analysis

The statistical significance of expression levels determined by qRT-PCR analysis was calculated by the Mann–Whitney U test, the difference was considered significant at *p* < 0.05. ROC-AUC graphs were generated with easyROC curve analysis (ver. 1.3.1.) (http://www.biosoft.hacettepe.edu.tr/easyROC/, accessed on 05 February 2021). Optimal cut-off points were determined by ROC analysis based on the best balance of sensitivity and specificity. In respect of all tests, significance level was *p* < 0.05.

## 5. Conclusions

In conclusion, our pilot study identified 53 significantly DE circulating miRNAs in plasma samples of GBM patients using the nCounter Human v3 miRNA Panel of the NanoString System. Among these, 52 miRNAs were found to be upregulated whereas 1 was downregulated. To validate our results based on NanoString analysis, relative expression of some miRNAs was determined by RT-qPCR measurements using hsa-miR-16-5p as the reference miRNA. Expression of hsa-miR-433-3p, hsa-miR-195-5p, and hsa-miR-29a-3p was significantly upregulated compared with those in the control samples, while in the case of hsa-miR-362-3p and hsa-miR-133a-3p we could not detect any expression either in healthy control samples or in GBM patient samples. We also constructed ROC-AUC curves using expression data obtained from non-malignant and malignant samples for the three significantly differently expressed miRNAs. Hsa-miR-433-3p, hsa-miR-195-5p, and hsa-miR-29a-3p could discriminate patients with malignant tumors from patients with non-malignant masses with a power AUC of 0.98214, 0.9704, and 0.98214, respectively. Our functional annotation analysis showed that experimentally validated targets of DE miRNAs are key regulators of tumor formation, suggesting that circulating miRNAs might play an important pathophysiological role in the formation of different tumor types. A clear limitation of our study is the low sample size; however, we feel that our results warrant validation in a large cohort of GBM patients.

## Figures and Tables

**Table 1:** List of miRNAs with significantly different plasma levels in GPs and HIs, listed according to their expression levels (log_2_FC) and showing their involvement in the formation of tumor types according to a manual search of the literature and the miR2Disease and miRCancer databases.

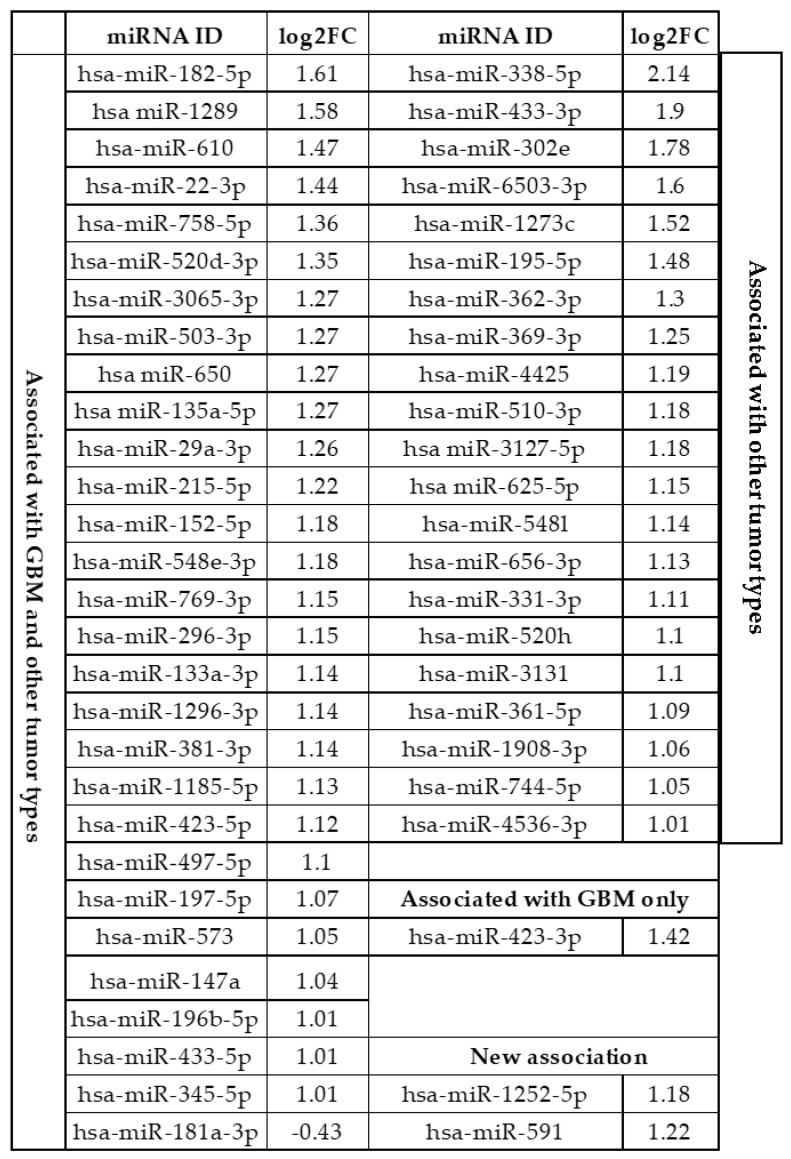

## Data Availability

Not applicable.

## Supplementary Materials

The following are available online at https://www.mdpi.com/article/10.3390/ijms22105058/s1. Figure S1: The minimum (a) and complete (b) networks built from miRNA-target gene and protein-protein interactome of the target genes coded proteins generated by the miRNet tool. Node sizes are proportional to their degree centrality values. (a) The miRNA nodes are lebeled by blue color, while proteins are reperesented by purple color. (b) The seed miRNAs (black nodes) are highlighted by a yellow rim.

## References

[1] Parsons D.W., Jones S., Zhang X., Lin J.C.-H., Leary R.J., Angenendt P., Mankoo P., Carter H., Siu I.-M., Gallia G.L. (2008). An Integrated Genomic Analysis of Human Glioblastoma Multiforme. Science.

[2] Van Meir E.G., Hadjipanayis C.G., Norden A.D., Shu H.-K., Wen P.Y., Olson J.J. (2010). Exciting New Advances in Neuro-Oncology: The Avenue to a Cure for Malignant Glioma. CA A Cancer J. Clin..

[3] Birkó Z., Nagy B., Klekner Á., Virga J. (2020). Novel Molecular Markers in Glioblastoma—Benefits of Liquid Biopsy. Int. J. Mol. Sci..

[4] Bartel D.P. (2004). MicroRNAs: Genomics, biogenesis, mechanism, and function. Cell.

[5] He L., Hannon G.J. (2004). MicroRNAs: Small RNAs with a big role in gene regulation. Nat. Rev. Genet..

[6] Zhao C., Tian F., Yu Y., Liu G., Zan L., Updike M.S., Song J. (2012). miRNA-dysregulation associated with tenderness variation induced by acute stress in Angus cattle. J. Anim. Sci. Biotechnol..

[7] Svoronos A.A., Engelman D.M., Slack F.J. (2016). OncomiR or Tumor Suppressor? The Duplicity of MicroRNAs in Cancer. Cancer Res..

[8] Xu S., Wang J., Ding N., Hu W., Zhang X., Wang B., Hua J., Wei W., Zhu Q. (2015). Exosome-mediated microRNA transfer plays a role in radiation-induced bystander effect. RNA Biol..

[9] Minciacchi V.R., Freeman M.R., Di Vizio D. (2015). Extracellular Vesicles in Cancer: Exosomes, Microvesicles and the Emerging Role of Large Oncosomes. Semin. Cell Dev. Biol..

[10] Kosaka N., Iguchi H., Yoshioka Y., Takeshita F., Matsuki Y., Ochiya T. (2010). Secretory mechanisms and intercellular transfer of MicroRNAs in living cells. J. Biol. Chem..

[11] Yang C., Wang C., Chen X., Chen S., Zhang Y., Zhi F., Wang J., Li L., Zhou X., Li N. (2012). Identification of seven serum microRNAs from a genome-wide serum microRNA expression profile as potential noninvasive biomarkers for malignant astrocytomas. Int. J. Cancer.

[12] Qu S., Guan J., Liu Y. (2015). Identification of microRNAs as novel biomarkers for glioma detection: A meta-analysis based on 11 articles. J. Neurol. Sci..

[13] Roth P., Wischhusen J., Happold C., Chandran P.A., Hofer S., Eisele G., Weller M., Keller A. (2011). A specific miRNA signature in the peripheral blood of glioblastoma patients. J. Neurochem..

[14] Ma C., Nguyen H.P.T., Luwor R.B., Stylli S.S., Gogos A., Paradiso L., Kaye A.H., Morokoff A.P. (2018). A comprehensive meta-analysis of circulation miRNAs in glioma as potential diagnostic biomarker. PLoS ONE.

[15] Fong M.Y., Zhou W., Liu L., Alontaga A.Y., Chandra M., Ashby J., Chow A., O’Connor S.T.F., Li S., Chin A.R. (2015). Breast-cancer-secreted miR-122 reprograms glucose metabolism in premetastatic niche to promote metastasis. Nat. Cell Biol..

[16] Okura H., Golbourn B.J., Shahzad U., Agnihotri S., Sabha N., Krieger J.R., Figueiredo C.A., Chalil A., Landon-Brace N., Riemenschneider A. (2016). A role for activated Cdc42 in glioblastoma multiforme invasion. Oncotarget.

[17] Zhao Y., Huang W., Kim T.M., Jung Y., Menon L.G., Xing H., Li H., Carroll R.S., Park P.J., Yang H.W. (2019). MicroRNA-29a activates a multi-component growth and invasion program in glioblasto-ma. J. Exp. Clin. Cancer Res..

[18] Luo X., Xu S., Zhong Y., Tu T., Xu Y., Li X., Wang B., Yang F. (2019). High gene expression levels of VEGFA and CXCL8 in the peritumoral brain zone are associated with the recurrence of glioblastoma: A bioinformatics analysis. Oncol. Lett..

[19] McDonald F.E., Ironside J.W., Gregor A., Wyatt B., Stewart M., Rye R., Adams J., Potts H.W. (2002). The prognostic influence of bcl-2 in malignant glioma. Br. J. Cancer.

[20] Da Huang W., Sherman B.T., Lempicki R.A. (2009). Systematic and integrative analysis of large gene lists using DAVID bioinformatics resources. Nat. Protoc..

[21] Mazurek M., Grochowski C., Litak J., Osuchowska I., Maciejewski R., Kamieniak P. (2020). Recent Trends of microRNA Significance in Pediatric Population Glioblastoma and Current Knowledge of Micro RNA Function in Glioblastoma Multiforme. Int. J. Mol. Sci..

[22] Grossman S.A., Ye X., Piantadosi S., Desideri S., Nabors L.B., Rosenfeld M., Fisher J. (2010). NABTT CNS Consortium Survival of Patients with Newly Diagnosed Glioblastoma Treated with Radiation and Temozolomide in Research Studies in the United States. Clin. Cancer Res..

[23] Klekner Á., Szivos L., Virga J., Árkosy P., Bognár L., Birkó Z., Nagy B. (2019). Significance of liquid biopsy in glioblastoma–A review. J. Biotechnol..

[24] Taft R.J., Pang K.C., Mercer T.R., Dinger M., Mattick J.S. (2009). Non-coding RNAs: Regulators of disease. J. Pathol..

[25] Hayes J., Peruzzi P.P., Lawler S. (2014). MicroRNAs in cancer: Biomarkers, functions and therapy. Trends Mol. Med..

[26] Di Leva G., Garofalo M., Croce C.M. (2014). MicroRNAs in Cancer. Annu. Rev. Pathol..

[27] Hsu S.-D., Lin F.-M., Wu W.-Y., Liang C., Huang W.-C., Chan W.-L., Tsai W.-T., Chen G.-Z., Lee C.-J., Chiu C.-M. (2010). miRTarBase: A database curates experimentally validated microRNA–target interactions. Nucleic Acids Res..

[28] Xie B., Ding Q., Han H., Wu D. (2013). miRCancer: A microRNA-cancer association database constructed by text mining on literature. Bioinformatics.

[29] He J., Jiang Y., Liu L., Zuo Z., Zeng C. (2021). Circulating MicroRNAs as Promising Diagnostic Biomarkers for Patients With Glioma: A Meta-Analysis. Front. Neurol..

[30] Rodrigues-Junior D.M., Pelarin M.F.A., Nader H.B., Vettore A.L., Pinhal M.A.S. (2021). MicroRNA-1252-5p Associated with Extracellular Vesicles Enhances Bortezomib Sensitivity in Multiple Myeloma Cells by Targeting Heparanase. OncoTargets Ther..

[31] Huh J.H., Kim T.H., Kim K., Song J.-A., Jung Y.J., Jeong J.-Y., Lee M.J., Kim Y.K., Lee D.H., An H.J. (2013). Dysregulation of miR-106a and miR-591 confers paclitaxel resistance to ovarian cancer. Br. J. Cancer.

[32] Singh S.K., Vartanian A., Burrell K., Zadeh G. (2012). A microRNA Link to Glioblastoma Heterogeneity. Cancers.

[33] Luo H., Zhang H., Zhang Z., Zhang X., Ning B., Guo J., Nie N., Liu B., Wu X. (2009). Down-regulated miR-9 and miR-433 in human gastric carcinoma. J. Exp. Clin. Cancer Res..

[34] Estep M., Armistead D., Hossain N., Elarainy H., Goodman Z., Baranova A., Chandhoke V., Younossi Z.M. (2010). Differential expression of miRNAs in the visceral adipose tissue of patients with non-alcoholic fatty liver disease. Aliment. Pharmacol. Ther..

[35] Wang W., Zhao L.J., Tan Y.-X., Ren H., Qi Z.-T. (2012). Identification of deregulated miRNAs and their targets in hepatitis B virus-associated hepatocellular carcinoma. World J. Gastroenterol..

[36] Sun S., Wang X., Xu X., Di H., Du J., Xu B., Wang Q., Wang J. (2016). MiR-433-3p suppresses cell growth and enhances chemosensitivity by targeting CREB in human glioma. Oncotarget.

[37] Hua D., Mo F., Ding D., Li L., Han X., Zhao N., Foltz G., Lin B., Lan Q., Huang Q. (2012). A Catalogue of Glioblastoma and Brain MicroRNAs Identified by Deep Sequencing. OMICS.

[38] Sun Y., Wang F., Wang L., Jiao Z., Fang J., Li J. (2017). MicroRNA-433 regulates apoptosis by targeting PDCD4 in human osteosarcoma cells. Oncol. Lett..

[39] Wang W.-Y., Lu W.-C. (2020). Reduced Expression of hsa-miR-338-3p Contributes to the Development of Glioma Cells by Targeting Mitochondrial 3-Oxoacyl-ACP Synthase (OXSM) in Glioblastoma (GBM). OncoTargets Ther..

[40] Kannan R., Lyon I., Baker N. (1980). Dietary control of lipogenesis in vivo in host tissues and tumors of mice bearing Ehrlich ascites carcinoma. Cancer Res..

[41] Jia Y., Tian Y., An S., Yang D. (2020). Effects of microRNA-195 on the Prognosis of Glioma Patients and the Proliferation and Apoptosis of Human Glioma Cells. Pathol. Oncol. Res..

[42] Shi L., Cheng Z., Zhang J., Li R., Zhao P., Fu Z., You Y. (2008). hsa-mir-181a and hsa-mir-181b function as tumor suppressors in human glioma cells. Brain Res..

[43] Yamada D., Fujikawa K., Kawabe K., Furuta T., Nakada M., Takarada T. (2018). RUNX2 Promotes Malignant Progression in Glioma. Neurochem. Res..

[44] Wu S., Wang S., Gao F., Li L., Zheng S., Yung W.K.A., Koul D. (2017). Activation of WEE1 confers resistance to PI3K inhibition in glioblastoma. Neuro Oncol..

[45] Li M., Xiao A., Floyd D., Olmez I., Lee J., Godlewski J., Bronisz A., Bhat K.P., Sulman E.P., Nakano I. (2017). CDK4/6 inhibition is more active against the glioblastoma proneural subtype. Oncotarget.

[46] Johansson M., Brännström T., Bergenheim A.T., Henriksson R. (2002). Spatial expression of VEGF-A in human glioma. J. Neuro Oncol..

[47] Benitez J.A., Ma J., D’Antonio M., Boyer A., Camargo M.F., Zanca C., Kelly S., Khodadadi-Jamayran A., Jameson N.M., Andersen M. (2017). PTEN regulates glioblastoma oncogenesis through chromatin-associated complexes of DAXX and histone H3.3. Nat. Commun..

[48] Ge S.X., Son E.W., Yao R. (2018). iDEP: An integrated web application for differential expression and pathway analysis of RNA-Seq data. BMC Bioinform..

