# Supplementary material for: Analysis of Circulating miRNA Profile in Plasma Samples of Glioblastoma Patients
Source: Int J Mol Sci. 2021 May 11;22(10):5058. doi: 10.3390/ijms22105058 (PMC8151942; doi:10.3390/ijms22105058)
Supplement: Supplementary file 1 [file ijms-22-05058-s001.zip › ijms-1178259-supplementary.pdf]

Supplementary Figure S1.

Figure S1. The minimum (**a**) and complete (**b**) networks built from miRNA-target gene and protein-protein interactome of the target genes coded proteins generated by the miRNet tool. Node sizes are proportional to their degree centrality values. a) The miRNA nodes are lebeled by blue color, while proteins are reperesented by purple color. b) The seed miRNAs (black nodes) are highlighted by a yellow rim.

(a)

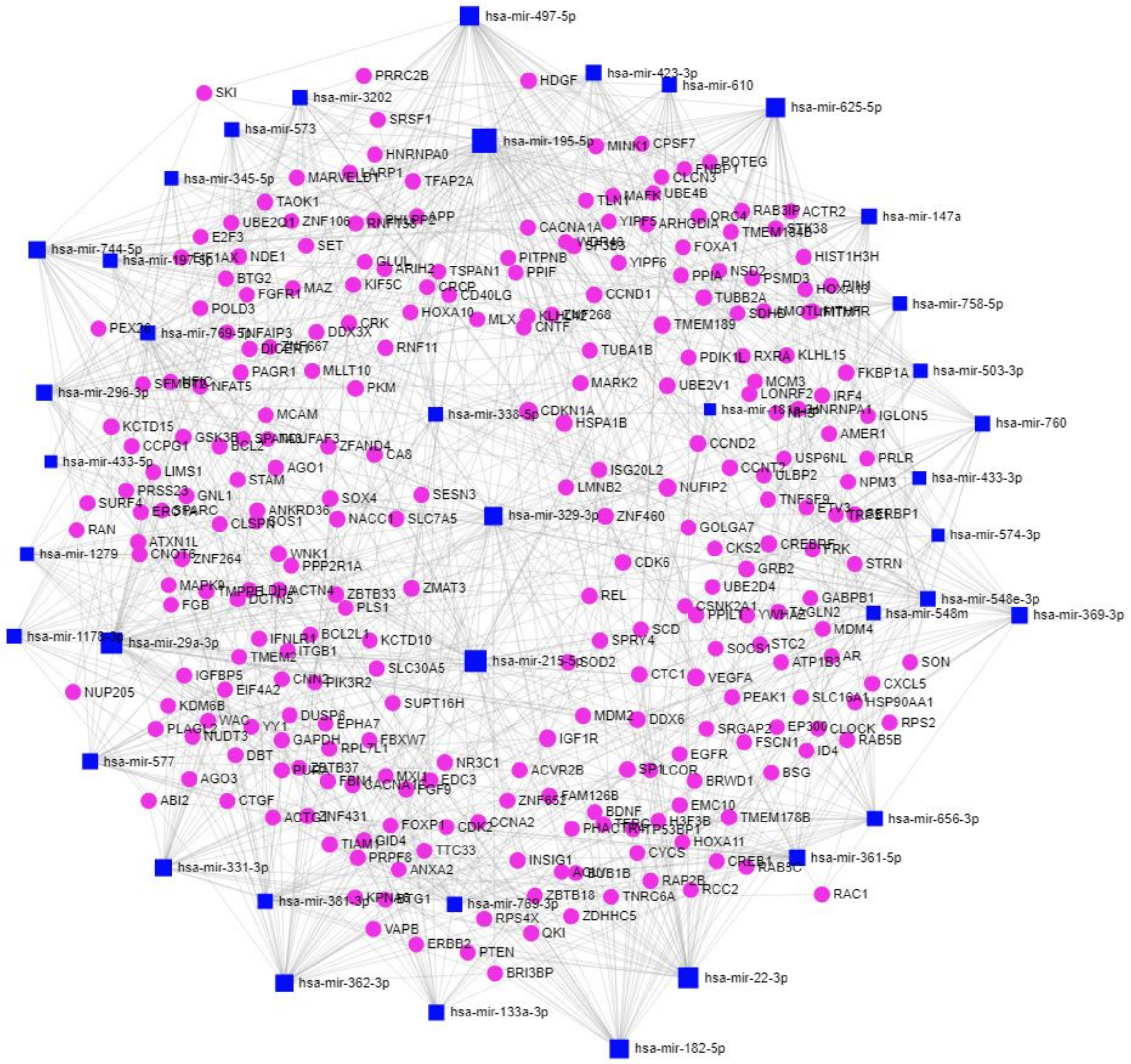

(b)

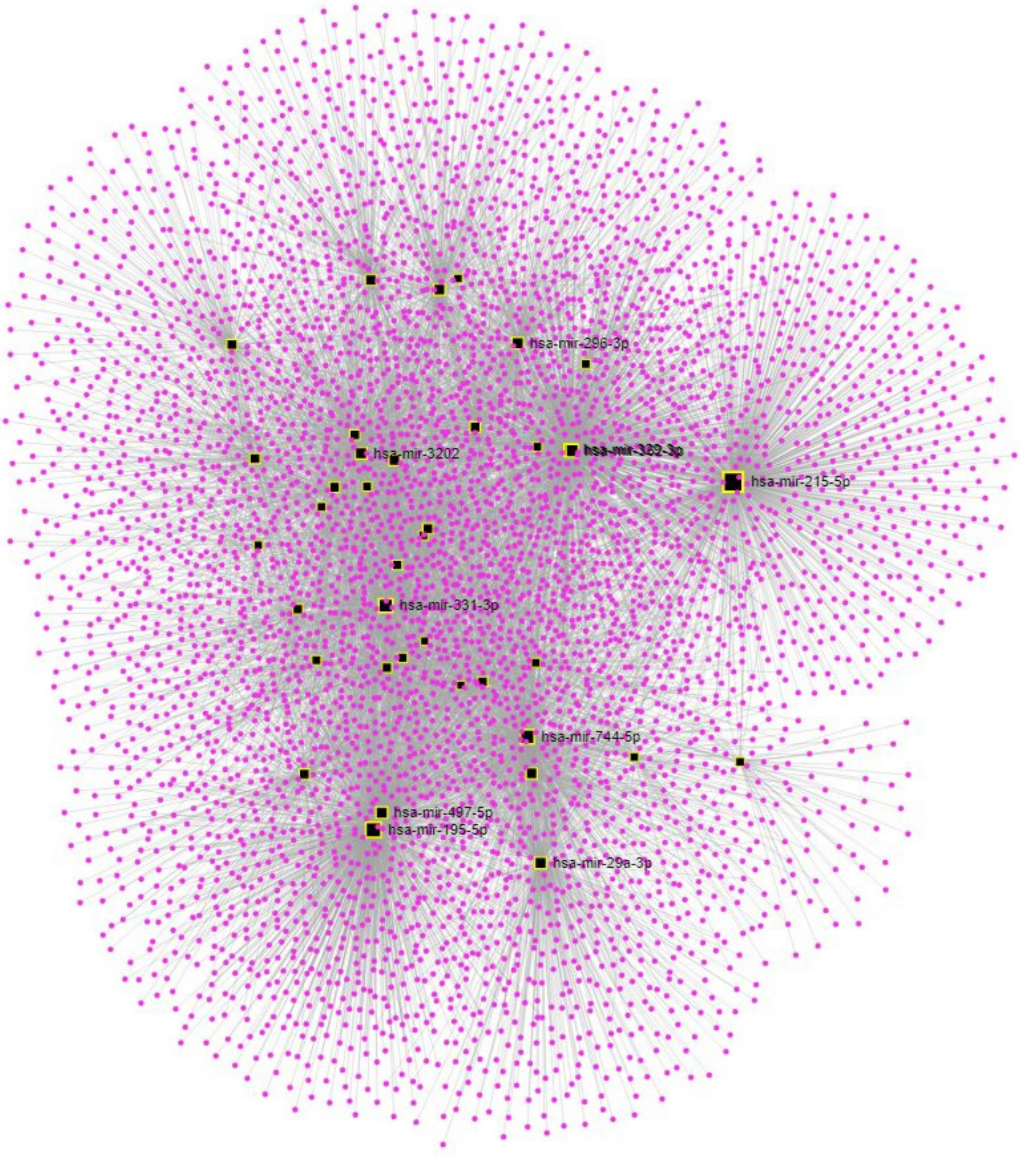
hsa-mir-296-3p
hsa-mir-3202
hsa-mir-215-5p
hsa-mir-331-3p
hsa-mir-744-5p
hsa-mir-497-5p
hsa-mir-195-5p
hsa-mir-29a-3p
